# Harnessing in-plane optical anisotropy in WS_2_ through ReS_2_ crystal

**DOI:** 10.1515/nanoph-2024-0672

**Published:** 2025-01-31

**Authors:** Soyeong Kwon, Tae Keun Yun, Peiwen J. Ma, SungWoo Nam

**Affiliations:** Department of Mechanical and Aerospace Engineering, University of California, Irvine, Irvine, CA 92697, USA; Department of Physics, Yonsei University, Seoul, 03722, South Korea; Department of Materials Science and Engineering, University of California, Irvine, Irvine, CA 92697, USA

**Keywords:** transition metal dichalcogenides, anisotropy, photoluminescence, charge transfer

## Abstract

In this work, we explore how the optical properties of isotropic materials can be modulated by adjacent anisotropic materials, providing new insights into anisotropic light-matter interactions in van der Waals heterostructures. Using a WS_2_/ReS_2_ heterostructure, we systematically investigated the excitation angle-dependent photoluminescence (PL), differential reflectance, time-resolved PL, and power-dependent PL anisotropy of WS_2_. Our findings reveal that the anisotropic optical response of WS_2_, influenced by the crystallographically low symmetry and unique dielectric environment of ReS_2_, significantly impacts both the optical and temporal behavior of WS_2_. We observed that the emission anisotropy increases with optical power density, highlighting that anisotropic localization of photo-generated carriers and subsequent charge transfer dynamics are key contributors to the polarization-sensitive optical response. These findings provide a framework for leveraging optical density-sensitive anisotropy mirroring to design advanced anisotropic optoelectronic and photonic devices.

## 1 Introduction

Two-dimensional (2D) group VI transition metal dichalcogenides (TMDs) exhibit direct bandgaps in their monolayer (1L) [1], [2] with near-unity quantum yield [3], even at high pump powers [4] and valley selective excitation at band edges [5], [6], [7], [8]. In their optical properties, excitons, quasiparticles consisting of electron-hole pairs, play a key role due to the reduced dielectric screening of Coulomb interactions, which allows a large exciton binding energy with excitonic states even at room temperature [9], [10], long diffusion length [11], [12], and formation of the various exciton species [13], [14]. Owing to the 3-fold rotational symmetry, group VI TMDs exhibit in-plane isotropic physical properties [15].

Anisotropic 2D materials can serve as templates for isotropic group VI TMDs to induce anisotropy in their physical properties [16]. Low-symmetry 2D materials, such as black phosphorus (BP) and transition metal monochalcogenides (TMMs), show anisotropy in their physical properties due to structural anisotropy from in-plane atom arrangement [17], [18]. However, these materials struggle with degradation under ambient conditions for BP [19] or low exfoliation yield for TMMs [20]. Group VII TMDs, such as ReS_2_, exhibit anisotropic physical properties arising from distorted 1T structure, characterized by Re–Re atoms in a zig-zag chain-like geometry along the *b*-axis. This unique arrangement leading to triclinic symmetry and the stability even under ambient conditions positions ReS_2_ as a promising 2D materials for harnessing polarization-sensitive optical excitation and emission [21], [22].

Furthermore, ReS_2_ can form type-II heterojunctions with most group VI TMDs [23], which are widely applied in optoelectronic devices [24], [25], [26], [27], memristors [28], and radiation enhancement [29] through efficient fast charge transfer between them [30]. While most studies focus on device applications leveraging efficient charge separation in type-II heterojunctions, the understanding of induced anisotropy of group VI TMDs remains limited. As ReS_2_ exhibits a limited quantum yield due to its indirect bandgap, integrating ReS_2_ with group VI TMD monolayers with direct bandgap enables the mirroring of its anisotropic optical responses while benefiting from the high quantum yield of the group VI materials [31].

In-plane anisotropic optical responses in ReS_2_ are attributed to its anisotropic optical oscillator strength relative to the excitation polarization, which leads to polarization-dependent photoemission, an anisotropic dielectric function, and a directional absorption coefficient [32]. Building on these anisotropic optical properties of ReS_2_, the anisotropic response of WSe_2_ on ReS_2_ has been explained by directional dielectric screening, which induces exciton localization due to the underlying ReS_2_ [33]. Additionally, although it has been suggested that the polarization behaviors of WS_2_ PL induced by ReS_2_ was due to anisotropic moiré potential leading to anisotropic absorption of bright excitons [34], previous work has primarily focused on demonstrating the anisotropy in photoluminescene (PL) and Raman scattering without systematic studies of the mechanisms of anisotropic optical responses.

In this work, we unveil the underlying mechanisms that explain how an isotropic material adjacent to anisotropic material can exhibit anisotropic optical properties. To comprehensively investigate the optical responses of individual WS_2_ and ReS_2_ layers, as well as their heterostructure regions, we employ PL, differential reflectance (d*R*), and time-resolved PL (TRPL) measurements at room temperature. Furthermore, under various optical pumping conditions, we track the optical anisotropy of WS_2_ on ReS_2_ and seek to understand how the optical anisotropy in the isotropic material emerges from the anisotropic material, particularly in terms of photo-generated charge carrier behaviors. This comprehensive analysis allows us to explore the role of optical excitation in inducing the optical anisotropy in WS_2_, originating from underlying ReS_2_ with intrinsic structural nonsymmetry.

## 2 Results and discussion

**Figure 1:**
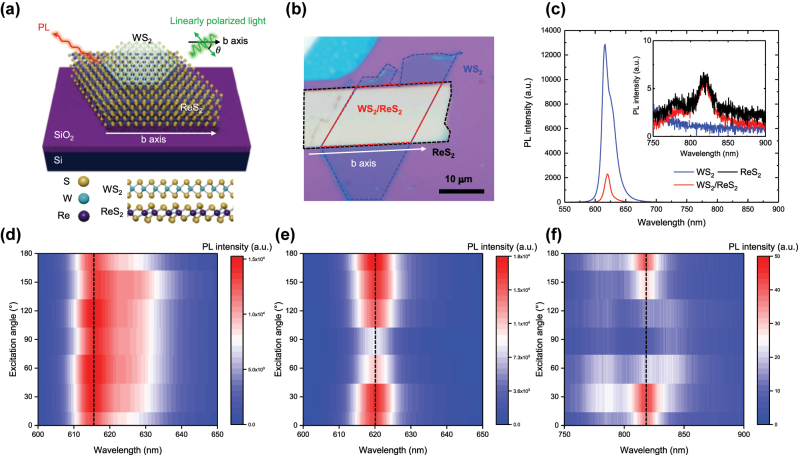
Structural Illustration and PL Characterization of WS_2_/ReS_2_ Heterostructure. (a) Schematic of fabricated WS_2_/ReS_2_ heterostructure and the side view of each layer. Linearly polarized light was excited with an excitation angle (*θ*), which is determined by the sample rotation. (b) Optical image of WS_2_/ReS_2_ heterostructure. (c) PL spectrum of WS_2_ (blue), ReS_2_ (black), and WS_2_/ReS_2_ (red) at excitation angle of 0°, with an inset showing magnified spectrum in the range of ReS_2_ emission. Excitation angle-dependent PL spectra of (d) WS_2_ and WS_2_/ReS_2_ in the ranges of (e) WS_2_ emission and (f) ReS_2_ emission.

To investigate the optical response of WS_2_ influenced by adjacent anisotropic materials, we prepared a heterostructure consisting of monolayer WS_2_ on multilayer ReS_2_. Figure 1(a) illustrates a schematic of the fabricated WS_2_/ReS_2_ sample on a SiO_2_/Si substrate, along with the configuration of the optical anisotropy measurement setup. The excitation angle (*θ*) is defined to be the angle between electric field of incident linearly polarized light and the *b*-axis of ReS_2_. Figure 1(b) shows the optical microscope image of fabricated WS_2_/ReS_2_ heterostructure. It was prepared by mechanical exfoliation of ReS_2_ flake onto SiO_2_/Si, followed by the dry transfer of an exfoliated WS_2_ flake on polydimethylsiloxane (PDMS) onto ReS_2_. As the natural cleavage axis of ReS_2_ corresponds to the *b*-axis during the mechanical exfoliation [22], [26], we could determine the *b*-axis, as illustrated in a white arrow.

To confirm the formation of the monolayer WS_2_ and multilayer ReS_2_ heterostructure, Raman spectroscopy was conducted (Supplementary Information, 1). In the WS_2_ region, the monolayer was identified by the Raman frequency difference (65 cm^−1^) between the in-plane *E* ^1^_2g_ mode at 349 cm^−1^ and the out-of-plane *A*_1g_ mode at 414 cm^−1^ [35]. In contrast, the multilayer ReS_2_ region exhibited multiple Raman peaks in the range of 100–250 cm^−1^, including *E*_g_ modes and an *A*_g_ – like mode. The peak position difference between Mode III (146 cm^−1^) and Mode I (134 cm^−1^), corresponding to 13 cm^−1^, indicates the presence of more than four layers of bulk ReS_2_. This is consistent with our atomic force microscopy (AFM) topography scan and height profiles showing 134 nm in the ReS_2_-only region and 136 nm in the WS_2_/ReS_2_ region (Supplementary Information, 2). In the overlapping WS_2_/ReS_2_ region, Raman spectra displayed characteristic peaks of both WS_2_ and ReS_2_ layers. A slight redshift of the ReS_2_ *A*_g_ – like mode (0.4 cm^−1^) in the heterostructure compared to the ReS_2_-only region suggests charge transfer induced by interlayer coupling [27], [36]. In the WS_2_/ReS_2_ heterostructure, the *A*_1g_ mode of WS_2_ was significantly suppressed due to its intrinsically low vibrational intensity and hybridization with the Raman modes of bulk ReS_2_ offering enhanced dielectric environment. The crystallographic orientation of ReS_2_, aligned along the *b*-axis, was confirmed by the enhanced ReS_2_ *A*_g_ – like mode *V* at 210 cm^−1^ under linearly polarized excitation light parallel to the *b*-axis (0°), compared to the excitation light perpendicular to the *b*-axis (90°).

We then investigated the anisotropic optical response of ReS_2_ and WS_2_ through excitation angle-dependent PL spectroscopy. The measurements were performed by rotating the sample while maintaining a fixed linear polarizer in the incident beam path and an analyzer in the collection beam path with cross-polarization configuration. At a *θ* of 0°, parallel to the *b*-axis (Figure 1(c)), the PL spectrum revealed dominant intralayer exciton resonance peaks from both WS_2_ and ReS_2_ layers.

The PL spectrum of WS_2_ was deconvoluted into two peaks: a neutral exciton at 615 nm and a trion at 630 nm, corresponding to the intrinsic n-type characteristics of WS_2_, with a trion binding energy of 42 meV [37]. Similarly, the PL spectrum of ReS_2_ exhibited two neutral exciton peaks at 780 nm and 817 nm, which result from the splitting of spin-degenerate exciton states [33], [38]. The indirect exciton states in ReS_2_ showed significantly lower PL intensities compared to the direct bandgap excitons of monolayer WS_2_, due to nonradiative recombination dominated by phonon-assisted scattering processes outside the light cone at room temperature. In the WS_2_/ReS_2_ heterostructure, excitons from both layers remained present, including red-shifted WS_2_ neutral exciton peak from 615 nm to 620 nm due to the dielectric screening effect of ReS_2_ [30], [33]. The intensities of intralayer excitons in heterostructure were noticeably suppressed, accompanied by a reduction in the trion PL intensity of WS_2_, suggesting charge transfer between the two layers [30]. The interlayer exciton, an electron–hole pair spatially separated in individual layers, was not observed due to the valley mismatch between the monolayer WS_2_ and ReS_2_ layers, which prevents the simultaneous transition required for an efficient interlayer exciton formation [39].

Interestingly, we observed that the excitonic species of WS_2_ at 620 nm, typically isotropic in nature exhibited anisotropic polarization-dependent PL intensity within the WS_2_/ReS_2_ heterostructure (Figure 1(d) and (e)). The highest PL intensities of WS_2_ and ReS_2_ at an excitation angle of 0°, along with their suppression at 90°, indicate that the distorted structure of ReS_2_ in contact with WS_2_ induces this anisotropic optical response (Figure 1(f)). By comparing the excitation angle-dependent PL intensities in individual WS_2_ and ReS_2_ layers, we confirmed that the isotropic emission of WS_2_ was modulated by the anisotropic optical response of ReS_2_ (Supplementary Information, 3).

**Figure 2:**
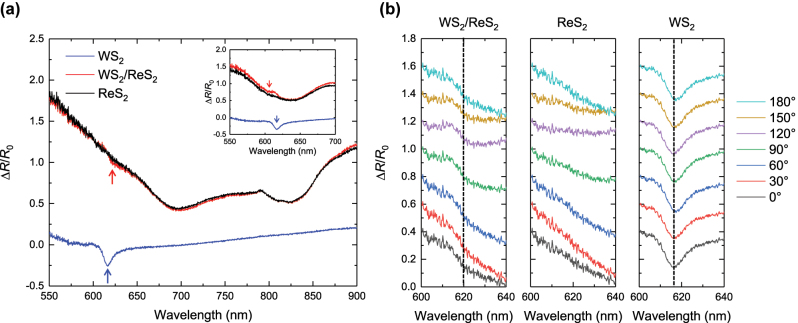
Differential Reflectance Characterization of WS_2_/ReS_2_ Heterostructure. (a) Differential reflectance of WS_2_ (blue), ReS_2_ (black), and WS_2_/ReS_2_ (red) at an excitation angle of 0°. The inset highlights the differential reflectance of each region in the wavelength range of 550–700 nm at an excitation angle of 90°. (b) Excitation angle-dependent differential reflectance spectra of WS_2_/ReS_2_, ReS_2_, and WS_2_ in the range of WS_2_ absorption.

To further substantiate our findings of anisotropic optical response of WS_2_ in the heterostructure region, we performed reflectance measurements using a broadband halogen lamp with linearly polarized white light and collected the reflected light intensities using a confocal optical microscope. The configuration was identical to that used for PL measurements, utilizing a linear polarizer and analyzer with sample rotation to change the excitation angle. The differential reflectance (Δ*R*/*R*_0_) was plotted, where *R* and *R*_0_ represent the reflectance spectra from the sample and the SiO_2_/Si substrate, respectively (Figure 2). As shown in Figure 2(a), the Δ*R*/*R*_0_ spectrum of WS_2_/ReS_2_ under an excitation angle of 0° exhibits a local minimum at 620 nm corresponding to WS_2_ exciton absorption resonance, which closely aligns with the PL peak position (Figure 1(c)). Additionally, heterostructure shows local minima at 690, 804, and 824 nm corresponding to the dips of ReS_2_.

From the excitation angle-dependent Δ*R*/*R*_0_ spectrum in the 600–640 nm range (Figure 2(b)), we observed that the WS_2_ exciton resonance dip feature at 620 nm displays periodicity at 90° intervals in WS_2_/ReS_2_. Notably, at 0°, the WS_2_ dip closely resembles that of ReS_2_, whereas at 90°, the dip resembles the dip from isotropic monolayer WS_2_ of which intrinsic dips lack the excitation angle dependence.

Assuming the dielectric contribution is dominated by the resonances of bound excitons, the broadening of the WS_2_ dip at 0° is attributed to strong exciton resonance hybridization resulting from crystallographic orientation-dependent dielectric constant of ReS_2_ as indicated by excitation angle-dependent Δ*R*/*R*_0_ spectrum (Supplementary Information, 4). Comparing the Δ*R*/*R*_0_ spectrum at an excitation angle of 90° to that at 0°, we observe a notable difference in the behavior of the WS_2_/ReS_2_ heterostructure, as illustrated in Figure 2(a) and its inset. At 0°, the dip in the Δ*R*/*R*_0_ spectrum of the heterostructure shows a smaller intensity than that of the individual ReS_2_ layer, indicating enhanced absorption of WS_2_ excitons. In contrast, at an excitation angle of 90°, the Δ*R*/*R*_0_ spectrum of ReS_2_ undergoes a general blue shift of its reflectance dip compared to excitation angle of 0°, leading to the dip of ReS_2_ in the WS_2_/ReS_2_ heterostructure moving closer to that of WS_2_. This results in an increase in its optical transition energy and a slight blue shift of WS_2_, along with higher intensity of Δ*R*/*R*_0_ indicating reduced absorption of WS_2_ excitons than under excitation at 0°. These observations suggest that the anisotropic dielectric environment of ReS_2_ significantly influences the WS_2_ exciton resonance in the heterostructure.

The PL spectra primarily reflect both direct and indirect transitions and are more sensitive to radiative recombination during transitions to the lowest energy state. In contrast, the Δ*R*/*R*_0_ spectrum, which resonates with direct transitions, provides more comprehensive information about charge transitions. This allows us to uncover the dielectric environment mirroring effect in WS_2_/ReS_2_, which is not as apparent in the PL spectra.

**Figure 3:**
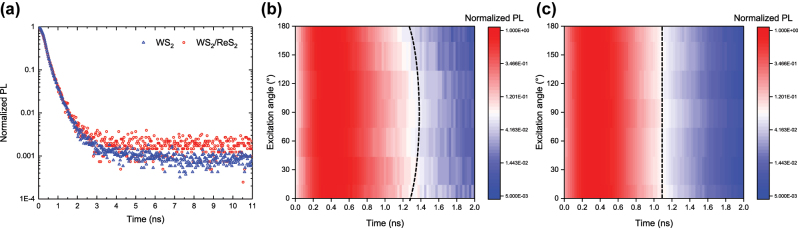
Time-Resolved PL Characterization of WS_2_/ReS_2_ Heterostructure. (a) Time-resolved PL decay characteristics in WS_2_ (blue) and WS_2_/ReS_2_ (red) at an excitation angle of 0°. Excitation angle-dependent decay characteristics in (b) WS_2_/ReS_2_ and (c) WS_2_.

To investigate the relationship between the optical anisotropy in temporal behaviors of excitons, we performed time-resolved photoluminescence (TRPL) measurements on WS_2_/ReS_2_ and WS_2_ using a time-correlated single photon counting (TCSPC) system, coupled with the same cross-polarization setup used in PL measurements. A bandpass filter (610 nm ± 10 nm) was placed in the light path of the TCSPC to isolate the radiative recombination of neutral WS_2_ excitons. In Figure 3(a), we compare the lifetime decay characteristics of WS_2_ and WS_2_/ReS_2_ under an excitation angle of 0°.

To ensure accurate analysis of the decay curve and precise determination of the PL lifetime, the decay curves for each region were normalized to their highest PL intensities and fitted with an exponential decay equation, *I* = *I*_0_e^−t/^ *^τ^*, to extract the exciton lifetime values, *τ*. Additionally, the time point where the normalized PL intensity equals to unity was used as the starting point for the decay analysis. The extracted lifetimes were *τ*_WS2/ReS2_ = 0.33 ± 0.003 ns, *τ*_WS2_ = 0.32 ± 0.004 ns, indicating negligible differences. Interestingly, lifetime decay characteristics at excitation angle of 90° reveal *τ*_WS2/ReS2_ increases to 0.38 ± 0.004 ns, in contrast to *τ*_WS2_ = 0.32 ± 0.004 ns, as shown in Figure 3(b) and (c) and Supplementary Information, 5. The previously reported PL lifetime of WS_2_ and transient absorption decay in ReS_2_/WS_2_ heterostructures, which were observed to be shorter than those of monolayer WS_2_ (within the 10–100 ps range) [26], [28], were not evident in our case.

Anisotropic lifetime of WS_2_ in the heterostructure region can be attributed to band anisotropy in combination with the electron–phonon scattering [40], [41], as well as more efficient charge mobility and exciton diffusion coefficient along the *b*-axis of ReS_2_. While more photocarriers are generated along the *b*-axis compared to the *a*-axis of ReS_2_ [40], [42], as indicated by higher PL intensities and absorption at 620 nm at an excitation angle of 0°, the enhanced charge transport efficiency along the *b*-axis results in faster charge recombination in WS_2_ on ReS_2_. Consequently, slightly shorter lifetime is justified to cool hot electrons via electron–phonon coupling along the *b*-axis. This observation aligns with angle-dependent relaxation times previously reported in SnS and BP, which exhibits optical anisotropy induced by orthorhombic structures [43], [44].

In addition, our observed lifetime anisotropy of WS_2_ in the WS_2_/ReS_2_ heterostructure region is consistent with previous time-resolved photoelectron emission microscopy results for WSe_2_ on ReS_2_ under controlled probe polarization [45]. In particular, the strong anisotropy of time constant suggests that interlayer charge transfer process is faster along the *b*-axis, due to faster evolution of excited electron distribution. The anisotropic lifetime of WS_2_ in the WS_2_/ReS_2_ suggests that the crystal orientation of ReS_2_ along the *a*- and *b*-axes may lead to variations in the band structure, including band dispersion flattening and the electron hopping behaviors [41], which can influence excitonic recombination lifetimes.

**Figure 4:**
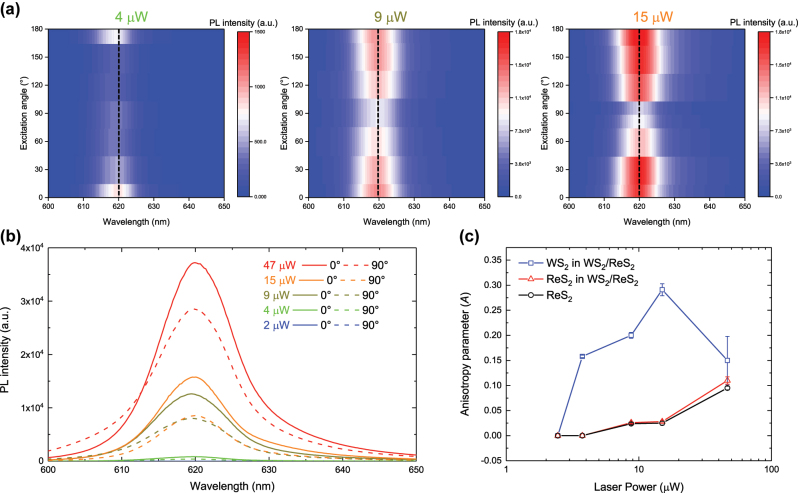
Laser Power-Dependent PL Characterization of WS_2_/ReS_2_ Heterostructure. (a) Excitation angle-dependent PL spectra of WS_2_/ReS_2_ in the WS_2_ emission range at laser powers of 4 μW, 9 μW, and 15 μW. (b) Power-dependent PL spectra at excitation angles of 0° and 90°, with laser power varying from 2 μW to 47 μW. (c) Anisotropy parameter of WS_2_ emission in WS_2_/ReS_2_ (blue), ReS_2_ emission in WS_2_/ReS_2_ (red), and ReS_2_ emission in ReS_2_ itself (black), as a function of laser power.

Lastly, we analyzed the power-dependent PL anisotropy by tracking the anisotropy parameter (*A*) defined as (*I*_max_ − *I*_min_)/(*I*_max_ + *I*_min_), as a function of laser power. *I*_max_ and *I*_min_ represent the PL peak intensity at 0° and 90°, respectively, with *I*_max_ showing the maximum intensity at 0° and *I*_min_ corresponding to the minimum intensity at 90°. First, as shown in Figure 4(a), the PL intensity contrast at different excitation angles increases with higher laser power. Figure 4(b) and (c) present the PL spectra at various laser powers for excitation angles of 0° and 90°, and the corresponding calculated *A* values, respectively. The anisotropy parameter *A* increases within the laser power range of 4–15 µW, while at a low power of 2 µW, the PL intensities decrease significantly due to insufficient optical excitation (Supplementary Information, 6 and 7). However, at the highest laser power of 47 µW, anisotropy parameter declines back to values observed in the 4 µW range, which implies the optical anisotropy influenced by optical densities of the photons.

The increase in *A* within the 4–15 µW laser power range can be attributed to not only the greater absorption of incident light enhancing the number of photo-generated carriers but also disparity in the amounts of the photo-generated carriers along the crystallographic axes. Specifically, at higher powers, the abundance of photo-generated carriers along the *b*-axis with excessive energy enhances wavefunction overlap, facilitating charge transfer from WS_2_ to ReS_2_ along the type-II band alignment [26], [28]. This effect is particularly pronounced when optical excitation occurs along the *b*-axis of ReS_2_ (0°), where optical oscillator strength is strongest originating from anisotropic electronic band structure. In this case, the effective charge transfer and separation of photocarriers become more significant, leading to stronger interlayer coupling between WS_2_ and ReS_2_. This coupling, combined with the inherent anisotropy of ReS_2_ due to its crystallographic axes, amplifies the optical anisotropy of WS_2_ at high optical densities.

The enhanced anisotropy of WS_2_ at high optical densities, resulting from an increased disparity in the amounts of photogenerated carriers depending on the optical excitation direction, is further supported by data in Supplementary Information, 8, where the integrated PL areas at 620 nm and 630 nm are compared. Each wavelength of the PL peak indicates radiative recombination of neutral exciton (620 nm) and trion (630 nm) [33], [37]. At an excitation angle of 0°, the proportion of the 630 nm area is higher at 15 µW than at 4 µW, suggesting an increase in trion population due to a higher optical doping carrier density. Additionally, as the excitation angle changes from 90° to 0°, the integrated area at 630 nm increases, indicating that the radiative recombination path of trions become more prominent due to anisotropically enhanced localizations of photo-generated carriers along the *b*-axis. The faster decay of WS_2_ neutral exciton in WS_2_/ReS_2_ at an excitation angle of 0° further supports the reduction in the integrated PL area of neutral excitons at the same angle.

On the other hand, the abnormal decrease in anisotropy at 47 µW can be explained by the increased role of electron–phonon coupling due to excessive thermal energy. Under these high power conditions, the trion population no longer increases as it did at 15 µW. Instead, the integrated 630 nm area remains comparable to that observed at 15 μW at excitation angle of 0°. The excessive thermal energy increases the likelihood of charge carriers being excited to higher energy states far from band edges, accompanied by phonon scattering. In heterostructures, enhanced electron–phonon coupling can lead to phonon softening, which induces weakening of interlayer coupling caused by an increased interlayer distance [46], [47], [48]. When the photon density of the laser exceeds the critical range, the increased interlayer distance impedes the efficient wavefunction overlap between the layers [49], [50], causing WS_2_ on ReS_2_ to lose its anisotropic optical response.

Furthermore, in contrast to the decrease in the anisotropy parameter of WS_2_ in the WS_2_/ReS_2_ heterostructure at the 47 µW, the anisotropy parameter of ReS_2_ in the heterostructure and ReS_2_ itself steadily increases. Therefore, we hypothesize that the anisotropic optical response of WS_2_, particularly induced by ReS_2_, arises from optical excitation, which generates photocarriers whose amounts vary with the optical excitation angle. At higher optical densities, disparity in population of localized photogenerated carriers increases, thereby enhancing the optical anisotropy in WS_2_ on ReS_2_.

## 3 Conclusions

In this study, we investigated how an isotropic material, WS_2_, adjacent to an anisotropic material, ReS_2_, can exhibit anisotropic optical responses. Through excitation angle-dependent PL, differential reflectance, time-resolved PL, and power-dependent PL anisotropy analyses, we demonstrated that the isotropic emission response of WS_2_ is influenced by the anisotropic optical properties of ReS_2_, which has crystallographically low symmetry. By comparing the excitation angle-dependent PL intensities of WS_2_ on ReS_2_ with those of individual WS_2_ and ReS_2_ layers, we confirmed that the anisotropic optical response of ReS_2_ modulates the optical behavior of WS_2_. In differential reflectance measurements, we observed that the anisotropic dielectric environment of ReS_2_ influences the WS_2_ exciton resonance. The shorter emission lifetime anisotropy in the WS_2_/ReS_2_ under excitation parallel to the *b*-axis indicates that the efficient charge mobility along the *b*-axis of ReS_2_ facilitates faster charge transfer. We identified that anisotropic charge transport originating from band structure anisotropy, along with electron–phonon scattering, should be carefully considered when interpreting the optical and temporal dynamics of WS_2_ excitons in WS_2_/ReS_2_ heterostructure.

The increasing anisotropy at higher optical photon densities suggests that the enhanced population of photo-generated carriers along the *b*-axis, along with their disparity, plays a key role in optical anisotropy. These results highlight the importance of carefully considering optical excitation, in conjunction with structural anisotropy, when interpreting optical responses in heterostructures consisting of isotropic and anisotropic material. This work provides valuable insights into harnessing anisotropic exciton states for the development of advanced anisotropic optoelectronic and photonic devices by symmetry breaking in 2D materials.

## 4 Materials and methods

### 4.1 Fabrication of WS_2_/ReS_2_ heterostructure

To assemble the WS_2_/ReS_2_ heterostructure, we used mechanical exfoliation and dry transfer. Multilayer ReS_2_ and monolayer WS_2_ were exfoliated from their bulk crystals (2D Semiconductors and HQ Graphene) with mechanical exfoliation (the Scotch tape method) onto SiO_2_/Si substrate and PDMS (Gel-Pak X4), respectively. We prepared WS_2_ with a cleaved edge along the isotropic crystallographic direction, exhibiting 60° angles at the corners, and ReS_2_ with a clearly defined *b*-axis along the anisotropic crystallographic direction. We stacked monolayer WS_2_ onto multilayer ReS_2_ on SiO_2_/Si with aligning the crystal axes, utilizing a rotation stage (Zaber XLSM025A) having an angular accuracy of up to 80 millidegrees (0.08°) to minimize atomic reconstruction between two layers. We used a transfer stage (Newport XYZ-PPP) inside a humidity-controlled glovebox to reduce the effects of adsorbates, contaminants, and oxidation of sample during transfer.

### 4.2 Optical and topographical characterizations

PL/Raman spectroscopy, differential reflectance spectroscopy, and time-resolved PL measurements were conducted using a customized confocal Raman microscope (NanoBase XPER Raman system) at room temperature. For PL/differential reflectance and Raman measurements, gratings with 300 lines per mm (lpmm) and 1800 lpmm were used, respectively, providing spectral resolutions of 0.9 nm and 4.3 cm^−1^. For excitation angle-dependent microscopic investigations, a linear polarizer was incorporated in the incident laser path to align the laser polarization direction with the *b*-axis of ReS_2_. An analyzer in a cross-polarization configuration was added, and the sample was manually rotated in 30° increments to adjust the excitation angle. Area mapping of optical responses was performed with lower than 1 µm spatial resolution using a galvo-mirror scanner, and area-averaged spectra were extracted to minimize discrepancies caused by surface roughness in single-point spectra.

For PL/Raman spectroscopy, a continuous-wave 532 nm green laser (Cobolt Tor) was used as the excitation source and emitted and scattered light was collected by the detector. Laser power was controlled using a continuous neutral density filter. For reflectance measurements, a broadband halogen lamp (U-LH100L-3) served as the excitation source. The sample was globally illuminated with white light, and the reflectance signal from the target area (selected via a galvo mirror) was collected using a confocal setup with 300 lpmm grating. Charge-coupled device camera (iVac 316, Andor) was employed for detection of PL/Raman and reflectance signals.

TRPL measurements were performed using a time-correlated single photon counting (TCSPC) system, consisting of a single-photon avalanche detector (PDM series, PicoQuant) and time-tagging electronics (TimeHarp 260, PicoQuant) with a timing resolution of 30 ps and 25 ps, respectively. A 510 nm ps pulsed diode laser (PDL 800D, PicoQuant) operating at an 80 MHz repetition rate was used for excitation. To isolate 510 nm excitation light and emission signals from neutral WS_2_ excitons, a 510 ± 10 nm bandpass filter was placed in the excitation path, and a 610 ± 10 nm bandpass filter was positioned before the detector.

Atomic force microscopy (AFM) (Vista One, Molecular Vista) was used for surface topography imaging and roughness analysis. A gold-coated silicon tip with a <35 nm radius (Q:NSC15/Cr-Au, MikroMasch) was employed for noncontact mode AFM scans. The scanning covered the sample area with a resolution of 512 pixels and a scan speed of 8 μm/s.

## Supplementary Material

Supplementary Material Details

## References

[1] Mak K. F., Lee C., Hone J., Shan J., Heinz T. F. (2010). Atomically thin MoS2: a new direct-gap semiconductor. *Phys. Rev. Lett*..

[2] Gutiérrez H. R. (2013). Extraordinary room-temperature photoluminescence in triangular WS 2 monolayers. *Nano Lett*..

[3] Lien D. H. (2019). Electrical suppression of all nonradiative recombination pathways in monolayer semiconductors. *Science*.

[4] Kim H., Uddin S. Z., Higashitarumizu N., Rabani E., Javey A. (2021). Inhibited nonradiative decay at all exciton densities in monolayer semiconductors. *Science*.

[5] Xiao D., Bin Liu G., Feng W., Xu X., Yao W. (2012). Coupled spin and valley physics in monolayers of MoS 2 and other group-VI dichalcogenides. *Phys. Rev. Lett*..

[6] Zeng H., Dai J., Yao W., Xiao D., Cui X. (2012). Valley polarization in MoS 2 monolayers by optical pumping. *Nat. Nanotechnol*..

[7] Mak K. F., He K., Shan J., Heinz T. F. (2012). Control of valley polarization in monolayer MoS2 by optical helicity. *Nat. Nanotechnol*..

[8] Cao T. (2012). Valley-selective circular dichroism of monolayer molybdenum disulphide. *Nat. Commun.*.

[9] He K. (2014). Tightly bound excitons in monolayer WSe2. *Phys. Rev. Lett*..

[10] Chernikov A. (2014). Exciton binding energy and nonhydrogenic Rydberg series in monolayer WS2. *Phys. Rev. Lett*..

[11] Kulig M. (2018). Exciton diffusion and halo effects in monolayer semiconductors. *Phys. Rev. Lett*..

[12] Cadiz F. (2018). Exciton diffusion in WSe2 monolayers embedded in a van der Waals heterostructure. *Appl. Phys. Lett*..

[13] Mak K. F. (2013). Tightly bound trions in monolayer MoS 2. *Nat. Mater*..

[14] You Y., Zhang X. X., Berkelbach T. C., Hybertsen M. S., Reichman D. R., Heinz T. F. (2015). Observation of biexcitons in monolayer WSe 2. *Nat. Phys*..

[15] Rasmussen F. A., Thygesen K. S. (2015). Computational 2D materials database: electronic structure of transition-metal dichalcogenides and oxides. *J. Phys. Chem. C*.

[16] Neupane G. P., Zhou K., Chen S., Yildirim T., Zhang P., Lu Y. (2019). In-Plane Isotropic/Anisotropic 2D van der Waals Heterostructures for Future Devices. *Small*.

[17] Liu H. (2014). Phosphorene: an unexplored 2D semiconductor with a high hole mobility. *ACS Nano*.

[18] Gomes L. C., Carvalho A. (2015). Phosphorene analogues: isoelectronic two-dimensional group-IV monochalcogenides with orthorhombic structure. *Phys. Rev. B:Condens. Matter Mater. Phys.*.

[19] Favron A. (2015). Photooxidation and quantum confinement effects in exfoliated black phosphorus. *Nat. Mater*..

[20] Sarkar A. S., Stratakis E. (2020). Recent advances in 2D metal monochalcogenides. *Advanced Science*.

[21] Lin Y. C. (2015). Single-layer ReS2: two-dimensional semiconductor with tunable in-plane anisotropy. *ACS Nano*.

[22] Chenet D. A. (2015). In-plane anisotropy in mono- and few-layer ReS2 probed by Raman spectroscopy and scanning transmission electron microscopy. *Nano Lett*..

[23] Jiang Y., Chen S., Zheng W., Zheng B., Pan A. (2021). Interlayer exciton formation, relaxation, and transport in TMD van der Waals heterostructures. *Light: Sci. Appl.*.

[24] Varghese A. (2020). Near-direct bandgap WSe2/ReS2 type-II pn heterojunction for enhanced ultrafast photodetection and high-performance photovoltaics. *Nano Lett*..

[25] Liu D. (2020). Synthesis of 2H-1T′ WS2-ReS2 heterophase structures with atomically sharp interface via hydrogen-triggered one-pot growth. *Adv. Funct. Mater*..

[26] Tang Y. (2020). Distinctive interfacial charge behavior and versatile photoresponse performance in isotropic/anisotropic WS2/ReS2Heterojunctions. *ACS Appl. Mater. Interfaces*.

[27] Zhao M. (2016). Interlayer coupling in anisotropic/isotropic van der Waals heterostructures of ReS2 and MoS2 monolayers. *Nano Res*..

[28] Huang F. (2023). Controllable resistive switching in ReS2/WS2 heterostructure for nonvolatile memory and synaptic simulation. *Advanced Science*.

[29] Karmakar A. (2022). Dominating interlayer resonant energy transfer in type-II 2D heterostructure. *ACS Nano*.

[30] Zereshki P., Yao P., He D., Wang Y., Zhao H. (2019). Interlayer charge transfer in ReS2/WS2 van der Waals heterostructures. *Phys. Rev. B*.

[31] Mohamed N. B. (2018). Photoluminescence quantum yields for atomically thin-layered ReS2: identification of indirect-bandgap semiconductors. *Appl. Phys. Lett*..

[32] Zhong H. X., Gao S., Shi J. J., Yang L. (2015). Quasiparticle band gaps, excitonic effects, and anisotropic optical properties of the monolayer distorted 1T diamond-chain structures ReS2 and ReSe2. *Phys. Rev. B:Condens. Matter Mater. Phys.*.

[33] Usman A. (2022). Enhanced excitonic features in an anisotropic ReS2/WSe2 heterostructure. *Nanoscale*.

[34] Xie X. (2023). Anisotropic optical characteristics of WS2/ReS2 heterostructures with broken rotational symmetry. *Appl. Phys. Lett*..

[35] Zeng H. (2013). Optical signature of symmetry variations and spin-valley coupling in atomically thin tungsten dichalcogenides. *Sci. Rep.*.

[36] Luo Y., Su W., Chen F., Wu K., Zeng Y., Lu H. W. (2023). Observation of strong anisotropic interlayer excitons. *ACS Appl. Mater. Interfaces*.

[37] Plechinger G. (2015). Identification of excitons, trions and biexcitons in single-layer WS2. *Phys. Status Solidi – Rapid Res. Lett.*.

[38] Dhara A. (2020). Additional excitonic features and momentum-dark states in ReS2. *Phys. Rev. B*.

[39] Binder J. (2019). Upconverted electroluminescence via Auger scattering of interlayer excitons in van der Waals heterostructures. *Nat. Commun*..

[40] Li D. (2021). Giant transport anisotropy in revealed via nanoscale conducting-path control. *Phys. Rev. Lett*..

[41] Biswas D. (2017). Narrow-band anisotropic electronic structure of ReS2. *Phys. Rev. B*.

[42] Cui Q. (2015). Transient absorption measurements on anisotropic monolayer ReS2. *Small*.

[43] Ge S. (2015). Dynamical evolution of anisotropic response in black phosphorus under ultrafast photoexcitation. *Nano Lett*..

[44] Sun K. (2022). Dynamical response of nonlinear optical anisotropy in a tin sulfide crystal under ultrafast photoexcitation. *J. Phys. Chem. Lett.*.

[45] Qin Y. (2023). Ultrafast electronic dynamics in anisotropic indirect interlayer excitonic states of monolayer WSe2/ReS2 heterojunctions. *Nano Lett*..

[46] Ling X. (2016). Anisotropic electron-photon and electron-phonon interactions in black phosphorus. *Nano Lett*..

[47] Sinha S., Arora S. K., Wu H. C., Sathe V. G. (2021). Phonon scattering mechanism in van der Waals heterostructures comprising of MoS2 and WS2 nanosheets. *Mater. Today: Proc.*.

[48] Goo T. Characterization of polarization-And power-dependent excitons and trions in bulk ReS2. *Appl. Phys. Lett*..

[49] Garrity O., Brumme T., Bergmann A., Korn T., Kusch P., Reich S. (2024). Interlayer exciton-phonon coupling in MoSe2/WSe2 heterostructures. *Nano Lett*..

[50] Reho R., Botello-Méndez A. R., Sangalli D., Verstraete M. J., Zanolli Z. (2024). Excitonic response in transition metal dichalcogenide heterostructures from first-principles: Impact of stacking, twisting, and interlayer distance. *Phys. Rev. B*.

