# Supplementary material for: Harnessing in-plane optical anisotropy in WS2 through ReS2 crystal
Source: Nanophotonics. 2025 Jan 31;14(10):1553–61. doi: 10.1515/nanoph-2024-0672 (PMC12116260; doi:10.1515/nanoph-2024-0672)
Supplement: Supplementary file 1 — Supplementary Material Details [file j_nanoph-2024-0672_suppl_001.docx]

**Supplementary Materials**

**Harnessing in-plane optical anisotropy in WS_2_ through ReS_2_ crystal**

Soyeong Kwon^1^, Tae Keun Yun^2^, Peiwen J. Ma^1^, and SungWoo Nam^1,3*^

^1^Department of Mechanical and Aerospace Engineering, University of California, Irvine, Irvine, CA 92697, USA

^2^Department of Physics, Yonsei University, Seoul, 03722, South Korea

^3^Department of Materials Science and Engineering, University of California, Irvine, Irvine, CA 92697, USA

***Corresponding Author Email:** [sungwoo.nam@uci.edu](mailto:sungwoo.nam@uci.edu)

**Keywords**: Transition metal dichalcogenides, Anisotropy, Photoluminescence, Charge transfer

 **Supplementary Information 1**. (a) Raman spectra of WS_2_ (blue), ReS_2_ (black), and WS_2_/ReS_2_ (red) at excitation angle of 0° and (b) 90°.

**Supplementary Information 2**. (a) Atomic force microscopy topography scan image and height profiles along (b) line 1 and (c) line 2.

**Supplementary Information 3**. (a) PL spectrum of WS_2_ (blue), ReS_2_ (black), and WS_2_/ReS_2_ (red) at excitation angle of 90°, with an inset showing magnified spectrum in the range of ReS_2_ emission. Excitation angle-dependent PL spectra of (d) ReS_2_ in the range of ReS_2_ emission.


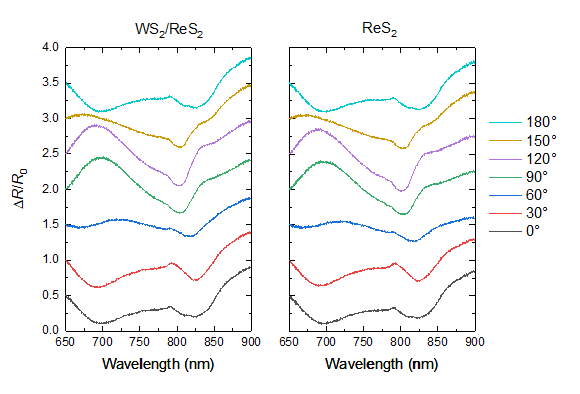


**Supplementary Information 4**. Excitation angle-dependent differential reflectance spectra of WS_2_/ReS_2_, ReS_2_ in the range of ReS_2_ absorption.


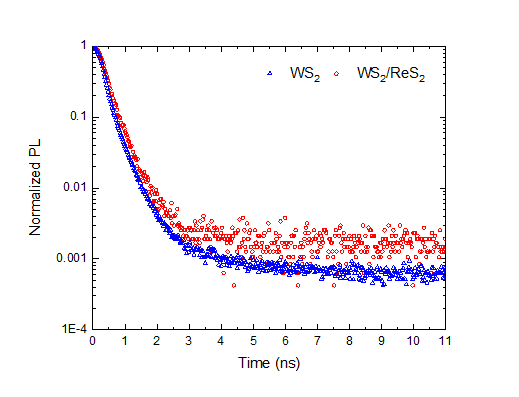


**Supplementary Information 5**. Time-resolved PL decay characteristics in WS_2_ (blue) and WS_2_/ReS_2_ (red) at an excitation angle of 90°.

**Supplementary Information 6**. Excitation angle (0° and 90°)-dependent PL spectra of WS_2_/ReS_2_ in the WS_2_ emission range at laser powers of (a) 2 μW and (b) 4 μW.

**Supplementary Information 7**. Excitation angle-dependent PL spectra of WS_2_/ReS_2_ in the WS_2_ emission range at laser powers of (a) 2 μW and (b) 47 μW.

**Supplementary Information 8**. Ratio of the integrated WS_2_ emission PL area, fitted with a Voigt function for each exciton (620 nm) and trion (630 nm) in WS_2_/ReS_2_ heterostructure at laser powers of (a) 4 μW, (b) 15 μW, and (c) 47 μW.
